# TRPM8 Channel Activation Induced by Monoterpenoid Rotundifolone Underlies Mesenteric Artery Relaxation

**DOI:** 10.1371/journal.pone.0143171

**Published:** 2015-11-23

**Authors:** Darizy Flavia Silva, Monica Moura de Almeida, Cinthia Guedes Chaves, Ana Letícia Braz, Maria Aparecida Gomes, Leidiane Pinho-da-Silva, Jorge Luiz Pesquero, Viviane Aguiar Andrade, Maria de Fátima Leite, José George Ferreira de Albuquerque, Islania Giselia Albuquerque Araujo, Xirley Pereira Nunes, José Maria Barbosa-Filho, Jader dos Santos Cruz, Nadja de Azevedo Correia, Isac Almeida de Medeiros

**Affiliations:** 1 Departamento de Bio-regulação, Universidade Federal da Bahia (UFBA), Salvador, Bahia, Brazil, 40110–902; 2 Departamento de Ciências Farmacêuticas, Universidade Federal da Paraíba (UFPB), João Pessoa, Paraíba, Brazil, 58059–900; 3 Departamento de Fisiologia e Biofísica, Universidade Federal de Minas Gerais (UFMG), Belo Horizonte, Minas Gerais, Brazil, 31270–901; 4 Núcleo de Estudos e Pesquisas de Plantas Medicinais da Universidade Federal do Vale do São Francisco, Petrolina, PE, Brasil, 56304–205; Indiana University School of Medicine, UNITED STATES

## Abstract

In this study, our aims were to investigate transient receptor potential melastatin-8 channels (TRPM8) involvement in rotundifolone induced relaxation in the mesenteric artery and to increase the understanding of the role of these thermosensitive TRP channels in vascular tissue. Thus, message and protein levels of TRPM8 were measured by semi-quantitative PCR and western blotting in superior mesenteric arteries from 12 week-old Spague-Dawley (SD) rats. Isometric tension recordings evaluated the relaxant response in mesenteric rings were also performed. Additionally, the intracellular Ca^2+^ changes in mesenteric artery myocytes were measured using confocal microscopy. Using PCR and western blotting, both TRPM8 channel mRNA and protein expression was measured in SD rat mesenteric artery. Rotundifolone and menthol induced relaxation in the isolated superior mesenteric artery from SD rats and improved the relaxant response induced by cool temperatures. Also, this monoterpene induced an increase in transient intracellular Ca^2+^. These responses were significantly attenuated by pretreatment with capsazepine or BCTC, both TRPM8 channels blockers. The response induced by rotundifolone was not significantly attenuated by ruthenium red, a non-selective TRP channels blocker, or following capsaicin-mediated desensitization of TRPV1. Our findings suggest that rotundifolone induces relaxation by activating TRPM8 channels in rat superior mesenteric artery, more selectively than menthol, the classic TRPM8 agonist, and TRPM8 channels participates in vasodilatory pathways in isolated rat mesenteric arteries.

## Introduction

The transient receptor potential (TRP) channels play various roles in the cardiovascular system, including: vasoconstriction, vasodilatation via the release of a peptide related to calcitonin gene, production of endothelial derived nitric oxide, muscle cell proliferation, myogenic responses, possible candidates for store-operated channels (SOCs) [1–2], receptor-operated channels (ROCs) [3–4] and stretch-activated ion channels (SAC) [2,5]. The TRP melastatin-8 channels (TRPM8) are the main ions channels that respond to cold stimuli. These channels were identified through electrophysiological studies of primary sensory neurons sensitive to cold [6–8]. Furthermore, TRPM8 channels are expressed differently in rat arteries and those channels allow for Ca^2+^ influx across the plasma membrane and sarcoplasmic reticulum mediated Ca^2+^ release, contributing to the vasomotor tone maintenance [9–10]. Additionally, TRPM8 channels are activated by cold temperatures (8–27°C) and cooling compounds such as menthol, eucalipthol and icilin [11–15].

Natural products have been important in identifying the functional role of TRP channels, especially the TRPM8 channel, in several biological tissues, demonstrating the importance of natural products in the assessment of TRP channel functions in the physiological systems [16]. Of the various constituents found in plants, the monoterpenes constitute the major component of essential oils derived from plants and have been reported in the literature as agonists or antagonists of TRP channels [17–20].

The literature has reported that rotundifolone, a monoterpenic ketone isolated from *Mentha x villosa* Hudson leaves, present analgesic activity [21], a spasmolytic effect on the ileum [22] and hypotension and bradycardia in anesthetized rats [23]. In previous studies, Guedes et al. [24, 25] observed that rotundifolone promoted hypotension and bradycardia in normotensives rats and induced endothelium-independent vasodilatation in the rat aorta. Additional, studies by Silva et al. [26] observed that rotundifolone induced vasodilatation in the superior mesenteric artery of Lyon normotensives (LN) through an endothelium-independent pathway, which involved the activation of large-conductance calcium-activated potassium channels (BK_Ca_) and inhibition of Voltage-gated calcium channels (Ca_v_ channels) [26]. However, the mechanism by which rotundifolone acts to influence the activity of these channels remains unclear.

The monoterpene, rotundifolone (C_10_H_15_O_2_), has a similar chemical structure to menthol (C_10_H_20_O) and is found in several species of the genus *Mentha* L.. Menthol is a known classic activator of TRPM8 channels, which can also be stimulated by other monoterpenes, such as: pulegol, eucalyptol and linalool [16]. In this study, we aimed to investigate if TRPM8 activation is involved in relaxation induced by rotundifolone in the mesenteric artery and to elucidate the role of these channels in vascular tissue. Furthermore, this study seeks to clarify the downstream signaling pathway involved following TRPM8 channel activation.

## Methods

### Animals welfare and Ethical Statement

Male Sprague Dawley (SD) rats (250–300g) were used in all experiments. Animals were housed under conditions of controlled temperature (21 ± 1°C) and lighting (lights on: 6:00AM –6:00PM). In addition, animals had free access to rat chow (PURINA-Brazil) and tap water *ad libitum*. The study was conducted in accordance with the Guide for the Care and Use of Laboratory Animals as adopted by the U.S. National Institutes of Health and it was approved by the Animal Care and Use Committees of the Federal University of Paraíba (CEUA/UFPB n° 1201/11 in 2011).

### Materials

#### Drugs

Acetylcholine chloride, L-phenylephrine chloride, ethylene glycol tetraacetic acid (EGTA), capsazepine, ruthenium red, menthol, capsaicin, SKF 96365, BCTC (4-(3-Chloro-2-pyridinyl)-N-[4-(1,1-dimethylethyl)phenyl]-1-piperazinecarboxamide) bovine serum albumin, hyaluronidase type II, chymopapain, DL-dithiothreitol and dimethyl sulfoxide (DMSO) were purchased from Sigma-Aldrich (Sigma Chemical Co., St. Louis, MO, USA). Collagenase type II was purchased from Worthington Biochemical Corporation (CLS2; Freehold, NJ, USA). TRPM8 antibody (ab3243) and secondary antibody (ab3243) were obtained from Abcam (Abcam, Cambridge, Massachusetts, USA). Stock solutions for capsazepine were prepared in DMSO. Rotundifolone was isolated and identified by the methods described by Almeida et al. 1996 [21]. Briefly, essential oil from *M*. *x villosa* was subjected to thin layer chromatography (Si-gel PF254, Merck, Darmstadt, Germany). The plates were developed three times with n-hexane as the solvent. Bands visible under a UV lamp were cut and extracted using CH_2_Cl_2_, and rotundifolone was obtained from the slower moving band with 99.9% spectroscopic purity. The monoterpenoid was solubilized in a mixture of water⁄cremophor. DMSO and cremophor at that concentration did not show any effect on our experiments

### Determination of TRPM8 expression by semi-quantitative PCR and western blotting in rat mesenteric artery

#### Semi-quantitative PCR

Superior mesenteric artery and prostate (as positive control) were removed and cleaned of adherent connective tissue and fat. Mesenteric artery was denuded of the endothelium layer. RNA was extracted from tissue using TRI Reagent following the recommended protocol. RNA concentration was determined using a biophotometer (A260 nm). Isolated RNA was reverse transcribed into cDNA using the high capacity cDNA reverse transcription kit (Invitrogen Life Technologies, Carlsbad, California, USA) according to the manufacturer’s instructions and was incubated in a thermal cycler (PTC-100 MJ Research Inc., Minneapolis, Minnesota, USA) at 40°C for 50 min, followed by 70°C for 15 min. The amplification reactions were performed in a final volume of 20 μl containing 10 mM Tris-HCl (pH 9.0), 0.1% Triton X-100, 75 mM KCl, 3.5 mM MgCl2, 0.2 mM desoxinucleotids (dATP/dTTP/dGTP/dCTP, Sigma Chemical Company, St Louis, Missouri, USA), 1U Platinum® Taq DNA polymerase (Invitrogen Life Technologies, Carlsbad, Califórnia, USA), 20 pmol sense (GCCCAGTGATGTGGACAGTA)/antisense (ATCTCCTCTGCGTTGTCGTT) primers (Exxtend, Campinas, Brazil) without (control) or with 1 or 2 μl reverse transcription reaction. The reactions were subjected to 35 cycles of 30 sec at 95°C (denaturation), 1 min at 55°C (annealing), and 1 min at 72°C (extension) followed by 10 min for the final extension in the thermal cycler. Electrophoresis on 10μl of each reaction was performed on a 4% polyacrylamide gel.

#### Western blotting

Mesenteric arteries were removed and prepared as described above. The mesenteric artery was homogenized in ice-cold RIPA lyses buffer containing a protease inhibitor cocktail (Roche, Mannheim, Baden-Württemberg, Germany). The homogenate was centrifuged at 4°C at 10,000 rpm for 10 min, the supernatant was collected, and the protein concentration was calculated using the Lowry method [27]. The protein samples were denatured and separated on a denaturing SDS/7.5% polyacrylamide gel, then transferred to a nitrocellulose membrane (PerkinElmer Inc, Waltham, Massachusetts, USA). The membrane was blocked with 5% (wt/vol) milk in TBS containing 0.1% Tween 20 (TBST) for 1 h at room temperature, followed by overnight incubation at 4°C with the specific primary rabbit polyclonal antibody against TRPM8 (1:1000 dilution; Abcam, Cambridge, Massachusetts, United States). After washing, the membrane was incubated with peroxidase-conjugated goat-anti-rabbit secondary antibody (1:3000 dilution; Abcam, Cambridge, Massachusetts, United States) at room temperature for 2 h. Protein loading was confirmed and normalized by comparing to β-actin levels.

### Preparation of isolated rat superior mesenteric artery rings

The superior mesenteric arteries were quickly removed of Sprague Dawley rats (250–300 g) as described by Silva and collaborators [26]. Briefly, mesenteric rings (1–2 mm length) were suspended by cotton threads in an organ bath containing 10 ml Tyrode’s solution and maintained at 37°C while gassed with a 95% O_2_ and 5% CO_2_ mixture (pH 7.4). Tyrode’s solution was composed of the following (in mM): NaCl 158.3; KCl 4.0; CaCl_2_· 2H_2_O 2.0; MgCl_2_· 6H_2_O 1.05; NaHCO_3_ 10.0; NaH_2_PO_4_· H2O 0.42 and Glucose 5.6 [28]. Rings were stabilized and a resting tension was applied at 0.75 g. Isometric tension was recorded by a force-displacement transducer (Miobath-4, WPI, Sarasota, FL, USA) coupled to an amplifier recorder (Transbridge-4; WPI, Sarasota, FL, USA). For most of experiments, the endothelium layer was removed by gently rubbing the intimal surface of the vessels with a cotton ball. Endothelial integrity was assessed qualitatively by the degree of relaxation caused by acetylcholine (10^−5^ M) in the presence of contractile tone induced by phenylephrine (10^−5^ M). Rings were considered to be endothelium-denuded when acetylcholine induced relaxant effects were less than 10%, while endothelium-intact was confirmed when the relaxant effects were greater than 90%.

#### Role of the TRPM8 channel on vasodilatation induced by rotundifolone and menthol

Endothelium-intact and -denuded superior mesenteric artery rings were contracted with phenylephrine (PHE 10^−5^ M), then challenged with either cumulative concentrations of rotundifolone (10^−7^ to 3x10^-3^ M) or menthol (10^−7^ to 10^−3^ M). To investigate the role of TRPM8 in vasodilatation caused by rotundifolone and menthol, arterial rings were pre-treated with ruthenium red (10^−5^ M), a non-selective TRP channels blocker, or with BCTC (2x10^-6^ M), a TRPM8 and TRPV1 channels blocker for a period of 30 minutes.

In order to exclude the participation of TRPV1 activity in rotundifolone- and menthol-mediated vasodilation, pretreatment with the specific agonist, capsaicin (10^−5^ M; 60 min) was performed to desensitize this channel, followed by PHE-induced contraction. This procedure was repeated until capsaicin-induced vasodilation was not observed.

#### Role of the Store-operated channel on vasodilatation induced by rotundifolone and menthol

To investigate the role of SOC in vasodilatation caused by rotundifolone and menthol, arterial rings were pre-treated with SKF 96365 (10^−5^ M), a SOC blocker, for a period of 30 minutes.

#### Effect of rotundifolone and menthol on the activation of TRPM8 channels by cold temperatures

In endothelium-denuded arterial rings, basal tension was reduced by temperature reduction in 3 stages: from 37°C to 25°C and to 18°C. After obtaining a control cooling-induced relaxation, the bath temperature was returned to 37°C, during which tone returned to the previous baseline. Following a 30 min equilibration period, rotundifolone or menthol was added to the bath for 15 min, after which the temperature was reduced from 37°C to 25°C and to 18°C. To further investigate the role of TRPM8 channels in the response induced by rotundifolone and menthol in cold temperatures conditions, EGTA 2mM, BCTC (2x10^-6^ M) or ruthenium red (10^−5^ M) were added to the bath at 37°C and equilibrated for 15 min before the addition of rotundifolone or menthol. The procedure described above was the repeated. Furthermore, the effects induced by cold temperature plus rotundifolone or menthol were analyzed after the TRPV1 desensitization with capsaicin (10^−5^ M).

### Preparation of vascular smooth muscle cells

Superior mesenteric arteries myocytes were enzymatically isolated from Sprague Dawley rats as described by Silva et al. [26]. Briefly, to obtain mesenteric myocytes for confocal Ca^2+^ imaging, the freshly dissected tissues were cut longitudinally and then incubated at 37°C in PSS containing 1 mg ⁄mL bovine serum albumin (BSA), 0.7 mg ⁄mL chymopapain and 1.0 mg ⁄mL dithiothreitol for 30 min. The tissue was then submitted to a low-Ca^2+^ (0.05 mM CaCl_2_) PSS, containing 1 mg ⁄mL BSA, 1 mg ⁄mL collagenase type II and 0.9 mg⁄mL hyaluronidase for 20 min. Single cells were obtained by gentle triturating through a Pasteur pipette, and aliquots of the cell suspension were placed in experimental chambers.

### Confocal Ca^2+^ imaging

Intracellular Ca^2+^ was measured in intact vascular myocytes with fluorescence laser scanning confocal microscopy as previously described [29–30]. For Ca^2+^ imaging, myocytes were incubated with fluo-4/AM (6 μM) (Invitrogen Life Technologies, Carlsbad, California, USA) for 40 minutes at 37°C, then coverslips containing the cells were transferred to a custom-built perfusion chamber on the stage of the BioRad MRC-1024 confocal microscope (BioRad, Hercules, California, United States) with the perfusion chamber maintained at room temperature. The cells were stimulated with rotundifolone (300 μM, 1mM and 3mM) or pretreated for 30 minutes with the EGTA (2mM) or capsazepine (20 μm) before rotundifolone addition. Fluo-4 fluorescence was monitored using 63 x oil immersed, 1.4 NA objective lens and images were collected at a rate of 1–5 frames/second. Changes in fluorescence (*F*) were normalized by the minimum fluorescence (*F*_0_) in each cell and were expressed as (*F*-*F*_0_/F_0_) × 100%.

### Data analysis

Data are presented as the mean ± S.E.M. and *n* represents a number of rings prepared from at least 3 different rats. Concentration-response curves to rotundifolone or menthol were based on the percent relaxation from the agonist-induced contraction. The curves were fitted using a variable slope sigmoid fitting routine in GraphPad Prism4 (San Diego, CA, USA). Statistical analyses were performed to compare the effects of rotundifolone or menthol (different concentrations) between groups of independent observations. Unpaired or paired Student's t-test, or one-way ANOVAs was used to compare 2, or 3 or more, groups respectively, with the addition of Bonferroni's multiple comparisons post-test.

## Results

### Identification of TRPM8 expression in rat vessels by semi-quantitative PCR and Western blotting

**Fig 1:**
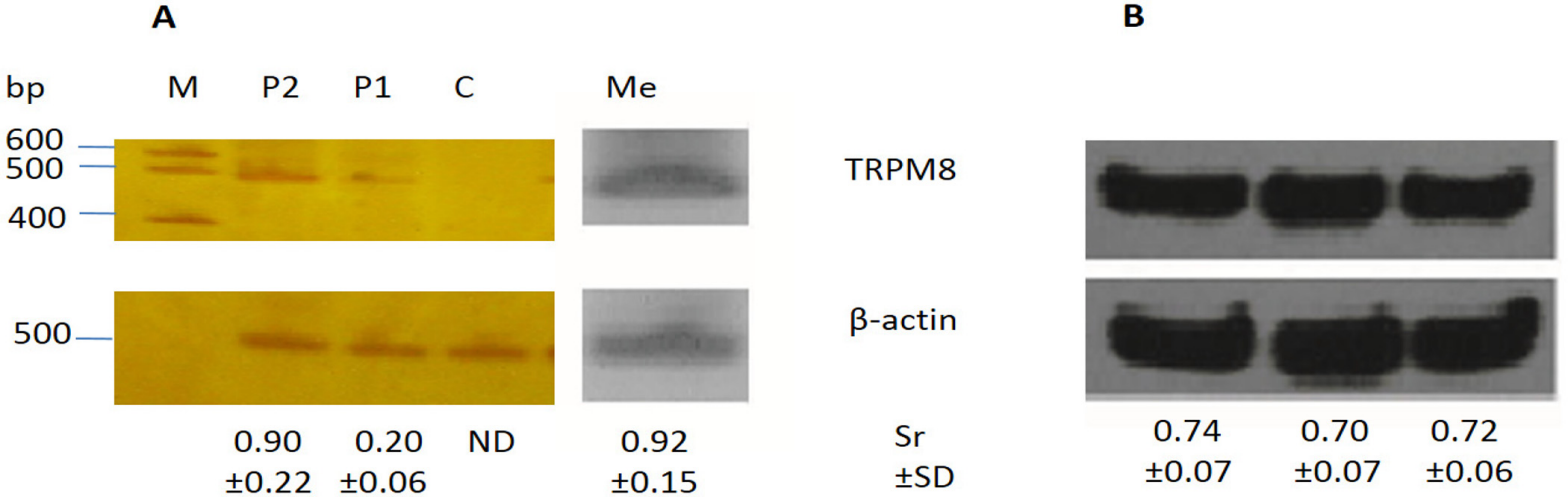
TRPM8 channels are present in rat superior mesenteric artery. (A) TRPM8 mRNA expression by semi-quantitative PCR in superior mesenteric artery with endothelium removed (n = 3). C, control; P1, 1 μL prostate RT reaction; P2, 2 μL prostate RT reaction, Me, 2 μL mesenteric artery RT reaction. (B) Western blot analysis of TRPM8 protein expression in rat vascular tissue. Molecular weight of TRPM8 was 125 KDa. Bottom panel: β actin used to demonstrate protein loading (at 42kDa).

Expression of TRPM8 receptor mRNA in rat superior mesenteric artery was observed by conventional semi-quantitative PCR. Fig 1A shows the amplified products at approximately 470 (TRPM8) and 500 (β-actin) base pairs generated after 35 cycles. The results were obtained in 3 separate experiments. Expression of TRPM8 channel proteins in vascular tissue was also examined using western blotting. The specific anti-TRPM8 antibody detected bands at approximately 125 kDa in 3 vascular tissue samples, (Fig 1B). These results are consistent with previous reports that TRPM8 can be expressed in rat mesenteric artery [10], as well as in other arteries, such as aorta and pulmonary artery [9].

### Relaxation induced by rotundifolone and menthol involve activation of TRPM8 channels

Initially, rotundifolone and menthol significantly relaxed the sustained contraction induced by phenylephrine (results in S1 Supplementary Material). Furthermore, there was no significant change in basal tone after rotundifolone exposure, contrary to what was observed with menthol, which induced contraction.

**Fig 2:**
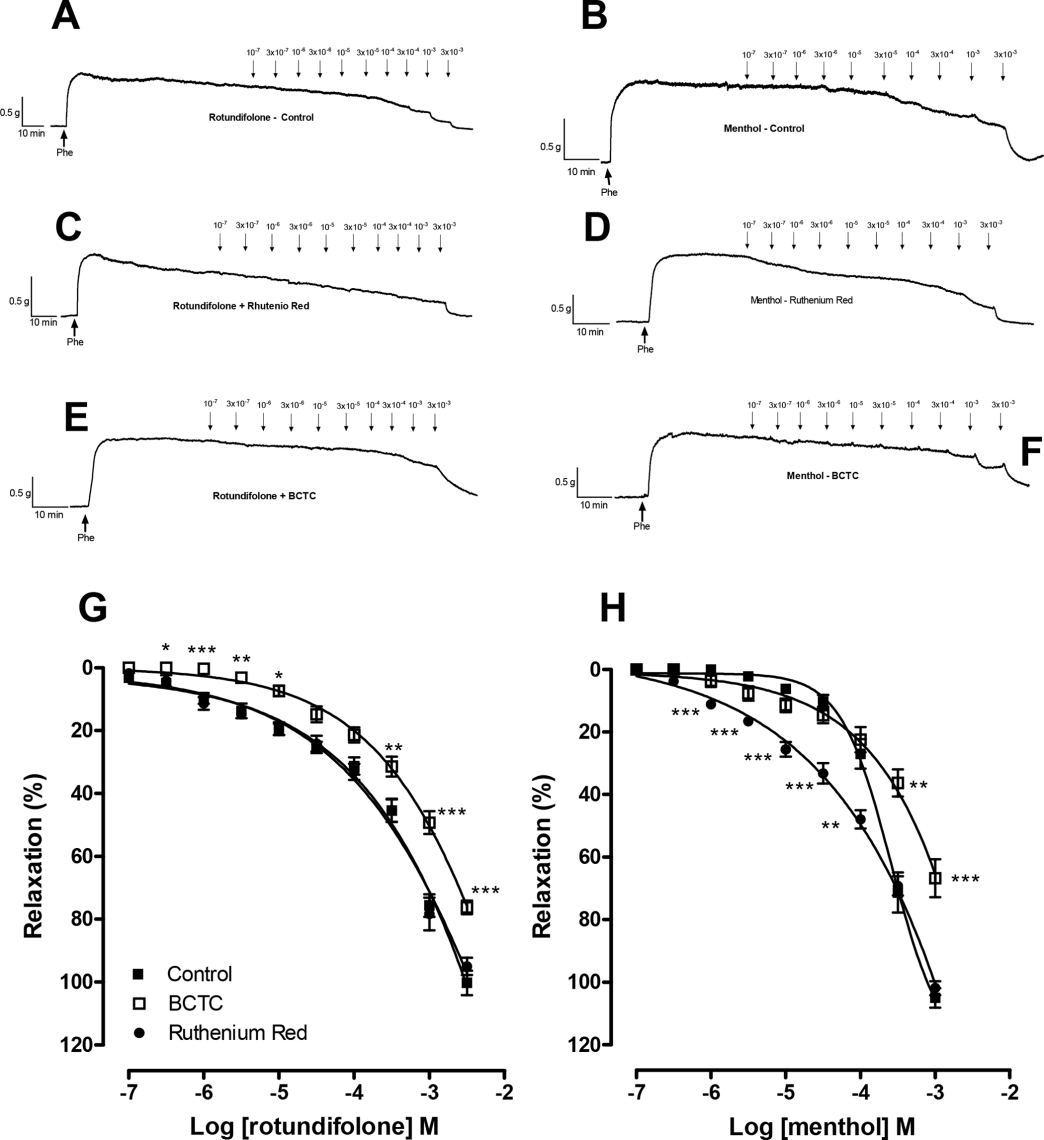
Influence of ruthenium red and BCTC in the menthol and Rotundifolone-induced dilation. Tension recording traces of relaxation responses induced by rotundifolone and menthol in denuded rat mesenteric arteries rings pre-contracted with phenylephrine in the absence (A, B) or in the presence of ruthenium red (10^−5^ M; C, D) or BCTC (2x10^-6^ M; E, F). Collective data are represented as bar graphs (G, H). Data are the mean ± SEM (n = 6). Data were examined using unpaired Student's t tests and one-way ANOVA followed by Bonferroni post-test.

In Fig 2C and 2G, endothelium-denuded rings pre-incubated for 30 min with ruthenium red (10^−5^ M), an organic polycationic compound known to block TRP channel family excluding among others the TRPM8 channels [31], did not alter the concentration–response curves for rotundifolone at any tested concentration [E_[3x10-3M]_ = 100.3 ± 3.9% (Control; Fig 2A and 2B), E_[3x10-3M]_ = 95.04 ± 3.92.7% (Ruthenium red), n = 6)]. However, menthol-induced relaxation was significantly potentiated in the presence of ruthenium red, as demonstrated by the leftward shift in the curve (Fig 2D and 2H). The participation of TRPM8 channels was investigated using BCTC, a TRPV1 and TRPM8 channel blocker [31–32]. In endothelium-denuded arterial rings, rotundifolone-mediated relaxation was significantly attenuated following BCTC pre-incubation (E_[3x10-3M]_ = 76.30 ± 2.15%, p<0.001, Fig 2E and 2G). Similar results were observed with menthol (E_[10-3M]_ = 66.77 ± 6.05%, p<0.001, Fig 2F and 2H).

### Effect of capsaicin-evoked desensitization of TRPV1 channels in relaxation induced by rotundifolone and menthol

**Fig 3:**
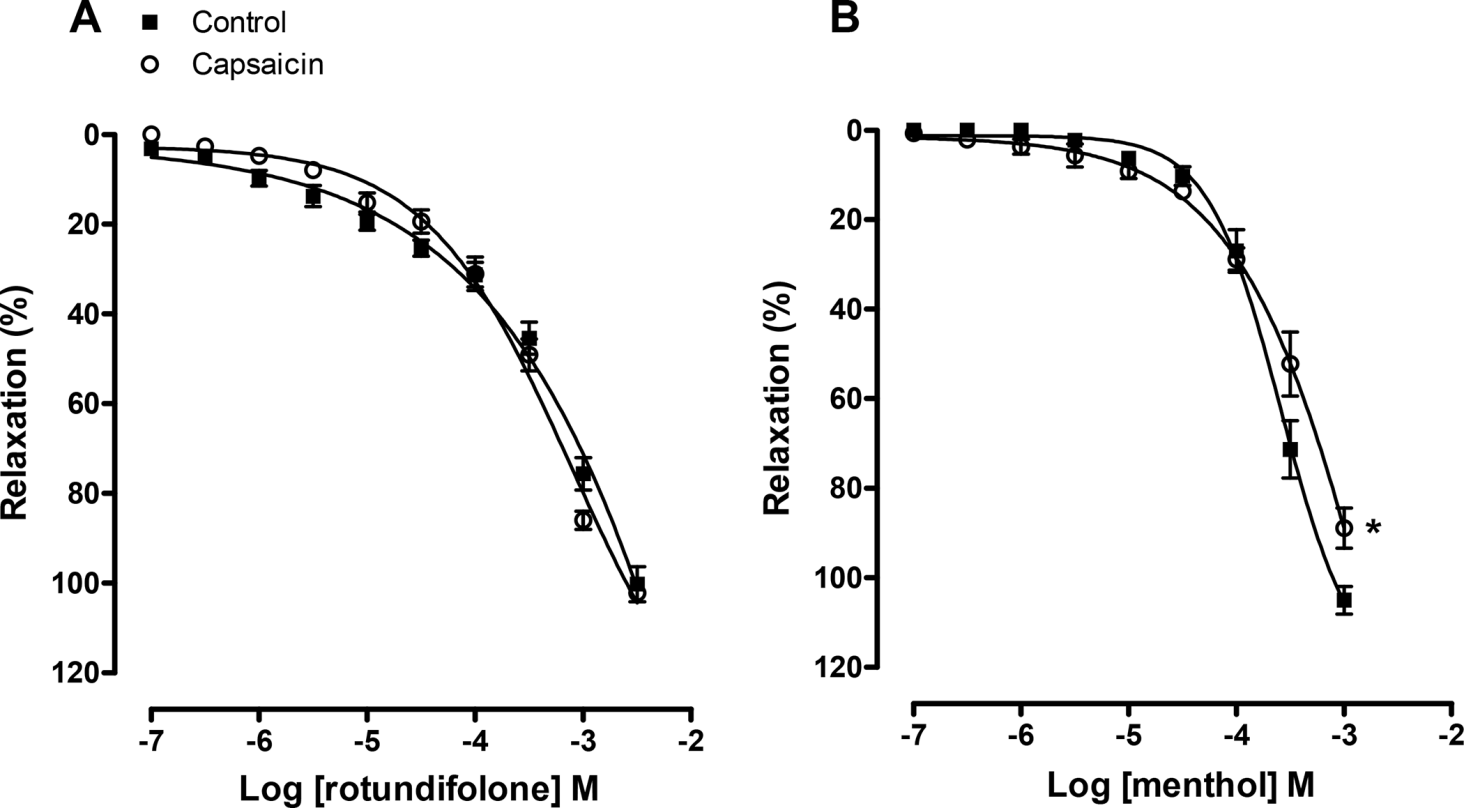
Vasorelaxation induced by rotundifolone is unaffected by capsaicin-evoked desensitization of TRPV1 channels. Relaxants responses induced by rotundifolone (A) and menthol (B) in denuded rat mesenteric arteries rings pre-contracted with phenylephrine in the presence of capsaicin (10^−5^ M). Data are the mean ± SEM (n = 6). The data were examined using unpaired Student's t tests and one-way ANOVA followed by the Bonferroni post-test.

Fig 3A shows that capsaicin-evoked desensitization of TRPV1 channels in endothelium-denuded arterial rings did not alter the rotundifolone concentration–response curves (E _[3x10-3M]_ = 102.25 ± 1.40%). On the other hand, menthol relaxation was attenuated as observed in E _[10-3M]_ values (88.96 ± 4.50%, p<0.01, Fig 3B), suggesting TRPV1 channel involvement in the mechanism of action of menthol, but not in rotundifolone.

### Effect of SOC inhibition in relaxation induced by rotundifolone and menthol

**Fig 4:**
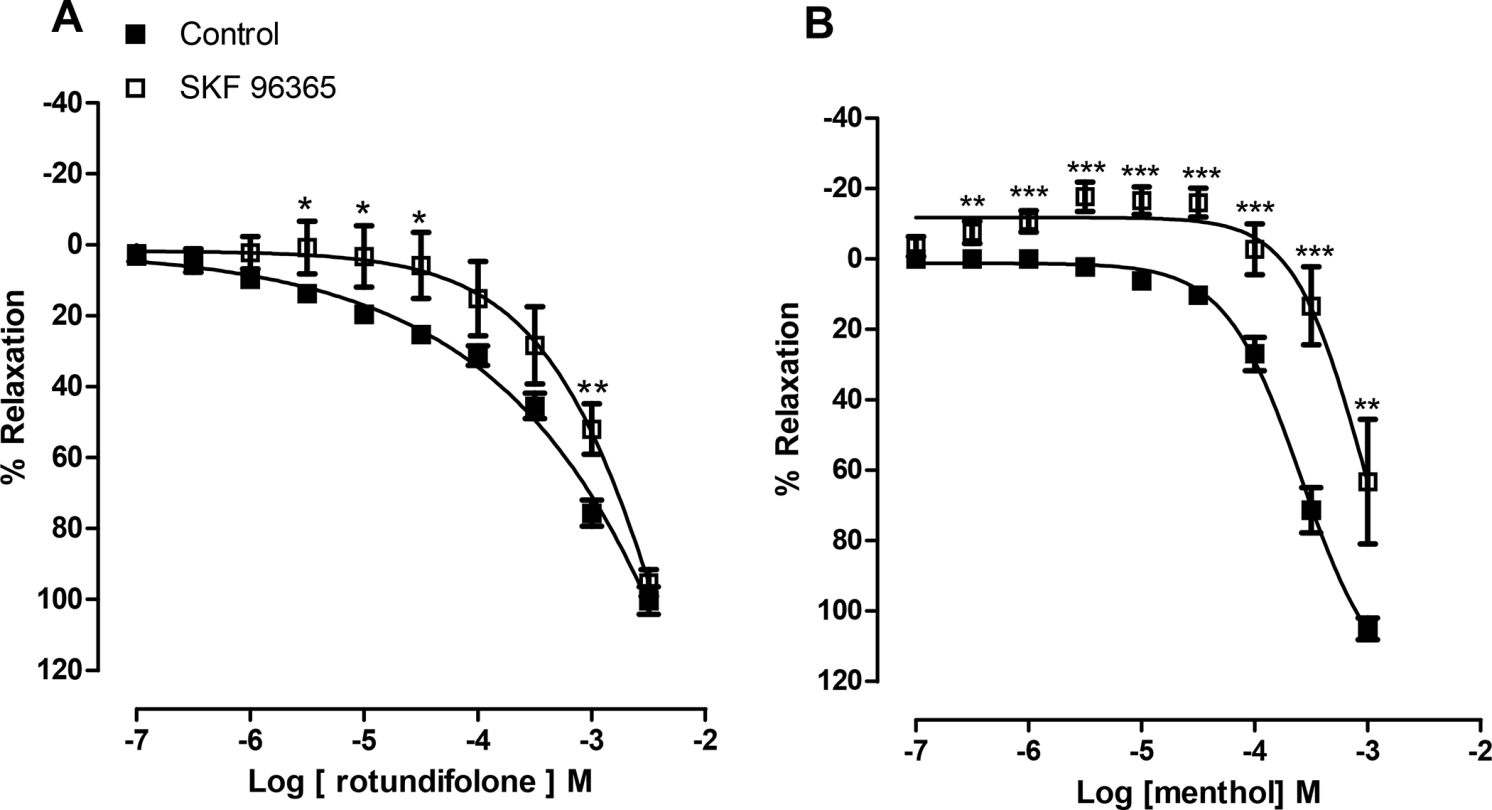
Vasorelaxation induced by rotundifolone is affected by SOC-inhibition. Relaxations responses induced by rotundifolone (A) and menthol (B) in denuded rat mesenteric arteries rings, pre-contracted with phenylephrine in the presence of SKF 96365 (10^−5^ M). Data are the mean ± SEM (n = 6). The data were examined using one-way ANOVA followed by the Bonferroni post-test.

Fig 4A shows that store operated channels (SOC) inhibition by SKF 96365 in endothelium-denuded arterial rings attenuated 4 of the 10 rotundifolone concentrations tested. On the other hand, menthol relaxation was significantly attenuated. Furthermore, at lower concentrations, menthol induced contractions in the presence of SKF 96365 (Fig 4B). These data suggests the involvement of SOC in the mechanism of action of rotundifolone and menthol.

### Effect of rotundifolone and menthol in rat mesenteric artery submitted to cold temperatures

**Fig 5:**
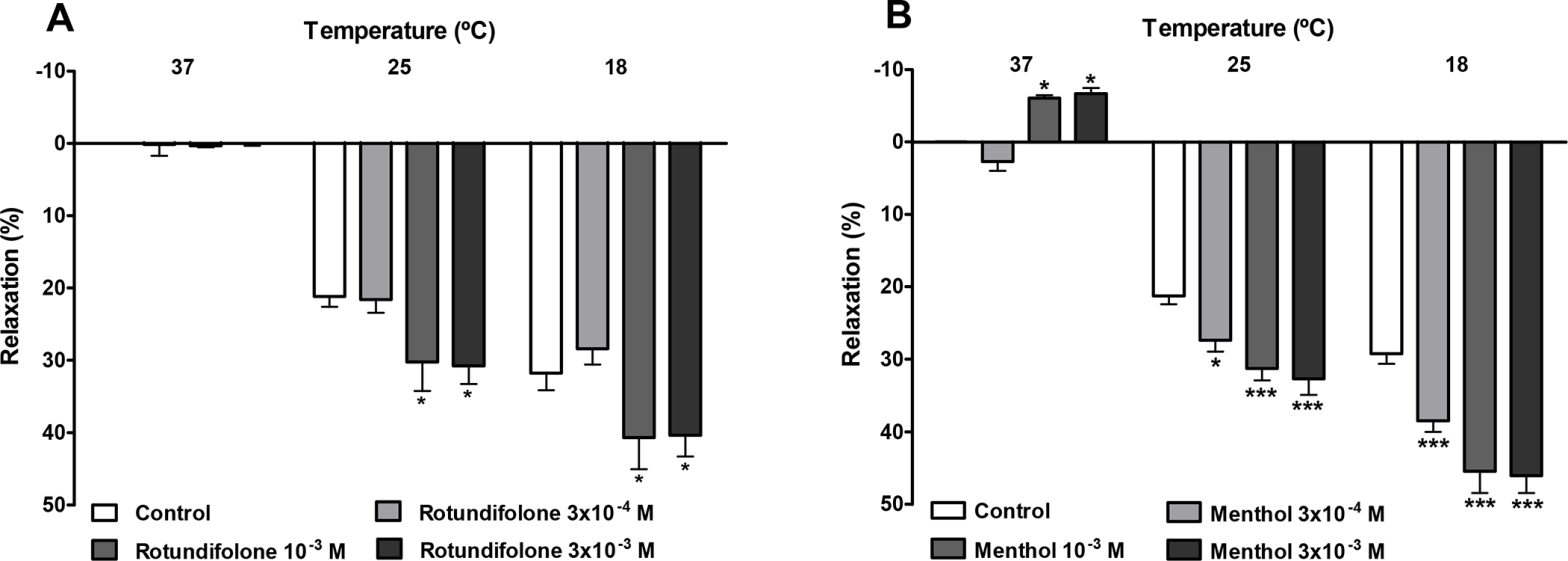
Rotundifolone and the cooling compound menthol enhanced the vasorelaxation effect induced by cold temperatures in mesenteric arteries. Bar graph summary showing that old temperature-induced vasodilatation is increased in the presence of rotundifolone (A) or menthol (3x10-4 M, 10–3 M and 3x10-3 M; n = 6) (B) in rat superior mesenteric arteries rings without vascular endothelium. Data are the mean ± SEM. The data were examined using one-way ANOVA followed by the Bonferroni post-test. *p < 0.05 and ***p < 0.001 rotundifolone or menthol vs control.

The effects of rotundifolone and menthol (positive control) on temperature-sensitive TRPM8 channels were investigated in endothelium-denuded rings submitted to cold temperatures. Fig 5A and 5B show that changes in bath temperature from 37°C to a temperature range that activates TRPM8 channels (25 or 18°C) induced a significant reduction in basal arterial tone (E_max_ = 21.25 ± 1.13 and 29.23 ± 1.36%, respectively, n = 6). This relaxing effect induced by cold temperature was significantly enhanced by both, rotundifolone (10^−3^ or 3x10^-3^ M) at 25°C (E_max_ = 30.25 ± 4.00 and 30.78 ± 2.47%, respectively, n = 6, p< 0.05) and 18°C (E_max_ = 40.68 ± 4.37 and 40.35 ± 2.94%, respectively, n = 6, p< 0.05). Similar results were observed with menthol (3x10^-4^, 10^−3^ or 3x10^-3^ M) at 25°C (E_max_ = 27.36 ± 1.56, 31.27 ± 1.64 and 32.70 ± 2.19%, respectively, n = 6, p < 0.05) and 18°C (E_max_ = 38.48 ± 1.55, 45.43 ± 3.01 and 48.08 ± 2.39%, respectively, n = 6, p < 0.05).

### Involvement of extracellular Ca^2+^ in menthol or rotundifolone induced relaxation of mesenteric artery rings submitted to cold temperatures

**Fig 6:**
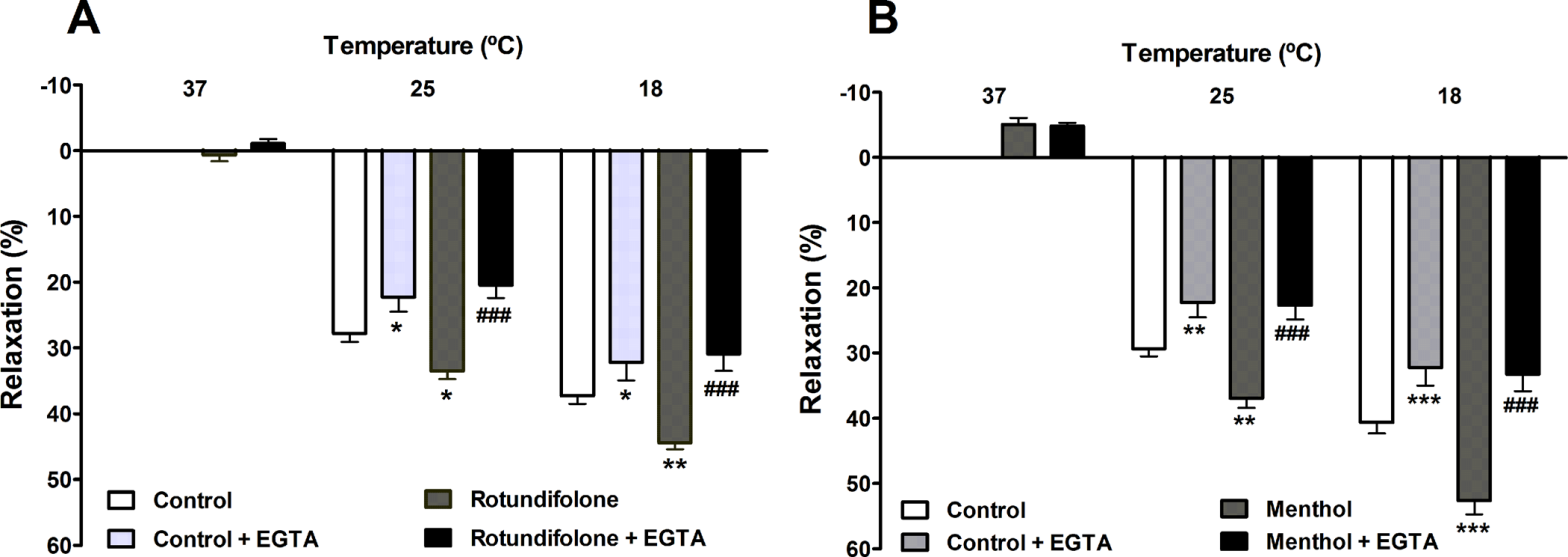
Rotundifolone and the cooling compound menthol enhanced the vasorelaxation effect induced by cold temperatures in rat mesenteric arteries by increasing calcium influx. Bar graph summary showing that the response induced by 3x10-3 M rotundifolone (A) or 10–3 M menthol (B) (n = 6) in the vasodilatation induced by cold temperatures in rat superior mesenteric arteries rings without vascular endothelium was attenuated in the presence of EGTA. Data are the mean ± SEM. The data were examined using one-way ANOVA followed by the Bonferroni post-test. *p < 0.05; **p < 0.01 and ***p < 0.001 rotundifolone or menthol vs control; ###p < 0.001 vs rotundifolone or menthol.

As shown in Fig 6A and 6B, the vasorelaxant effect induced by cold temperature (25 and 18°C) (E_max_ = 29.24 ± 1.16 and 40.66 ± 1.69%, respectively) was significantly attenuated by the removal of Ca^2+^ and the addition of EGTA in the bathing solution (Ca^2+^-free solution) (E_max_ = 22.26 ± 2.23 and 32.22 ± 2.76%, respectively). The additional relaxation effect induced by rotundifolone (3x10^-3^ M) in rings submitted to cold temperatures (25° and 18°C) (E_max_ = 30.50 ± 1.23 and 44.44 ± 0.96%, respectively) was also significantly attenuated when bathed in the Ca^2+^-free solution (E_max_ = 20.42 ± 1.97 and 30.90 ± 2.58%, respectively). Similar results were observed in arterial rings challenged with menthol and subjected to cool temperatures (25° and 18°C) before (E_max_ = 36.94 ± 1.46 and 52.62 ± 2.10%, respectively) and after addition of Ca^2+^-free solution (E_max_ = 22.60 ± 2.16 and 33.24 ± 2.61%, respectively).

### TRPM8 channels are involved in the relaxant effect induced by rotundifolone and menthol in arterial rings submitted to cold temperatures

**Fig 7:**
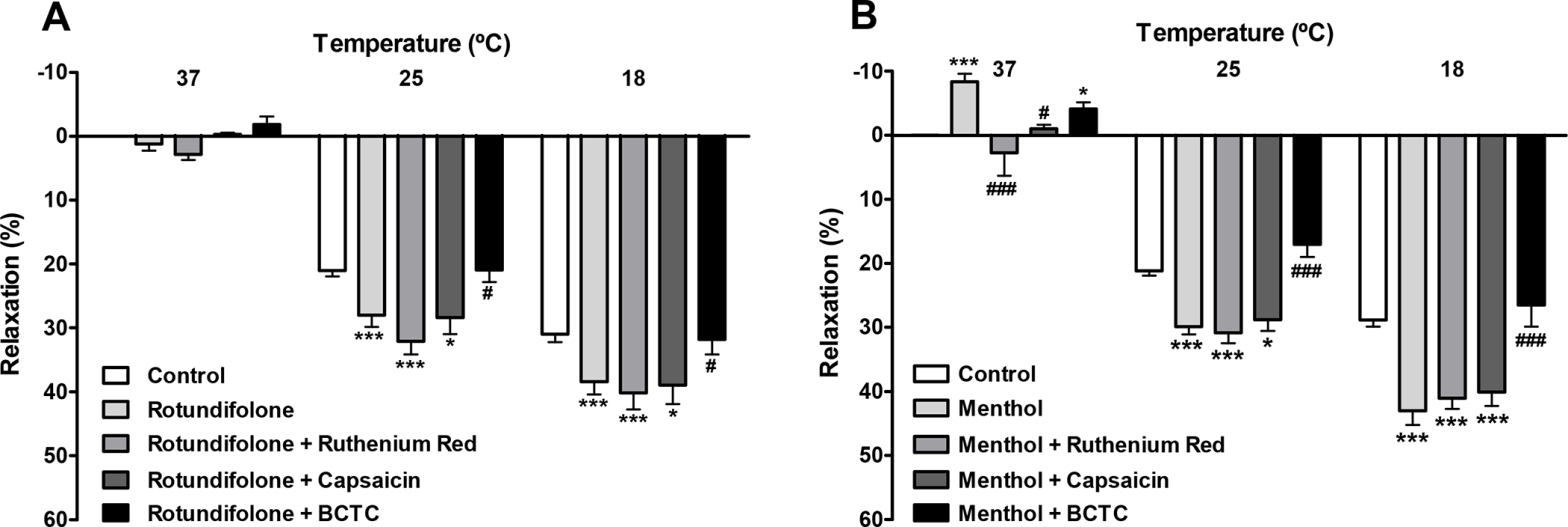
Vasorelaxation induced by rotundifolone and menthol in rat mesenteric arteries submitted to cold temperatures involves TRPM8 activation. Bar graph summary shows vasodilatation induced by (A) rotundifolone (3x10^-3^ M, n = 6) or (B) menthol (10^−3^ M, n = 6) in rat mesenteric arteries without vascular endothelium submitted to cold temperatures, was abolished in the presence of BCTC (2x10^-6^ M). Neither ruthenium red (10^−5^ M) nor capsaicin-evoked desensitization (10^−5^ M) of TRPV1 channels modified the relaxation induced by menthol or rotundifolone. Data are presented as the mean ± SEM. The data were examined using one-way ANOVA followed by the Bonferroni post-test. *p<0.05, **p<0.01 and ***p<0.001 vs control; #p< 0.05 and ###p<0.001 vs rotundifolone or menthol.

The additional relaxation induced by rotundifolone (3x10^-3^ M) in arterial rings subjected to cold temperatures of 25°C or 18°C (E_max_ = 28.01 ± 1.81 and 38.45 ± 1.98% respectively) was not accompanied by a significant change when in the presence of 10^−5^ M ruthenium red (E_max_ = 32.08 ± 2.05 and 40.18 ± 2.59%, respectively) or following TRPV1 channel desensitization with 10^−5^ M capsaicin (E_max_ = 28.40 ± 2.56 and 38.96 ± 2.98%, respectively). However, potentiation of the relaxation response induced by cold temperature following the addition of 3x10^-3^ M rotundifolone was significantly reduced in the presence of 2x10^-6^ M BCTC (E_max_ = 20.96 ± 1.89 and 31.84 ± 2.34%, respectively, Fig 7A). No significant change was observed in the relaxation effect of menthol (10^−3^ M; E_max_ = 29.87 ± 1.25 and 43.03 ± 2.22%, respectively) after pretreatment with ruthenium red (10^−5^ M; E_max_ = 30.85 ± 1.62 and 41.06 ± 1.66%, respectively) or capsaicin (10^−5^ M; E_max_ = 28.80 ± 1.75 and 40.10 ± 2.16%, respectively) in mesenteric artery rings subjected to the same cold condition described above. Similar to what was observed with rotundifolone, menthol–mediated relaxation also significantly decreased in the presence of 2x10^-6^ M BCTC (E_max_ = 17.05 ± 1.94 and 26.48 ± 3.39%, respectively). However, menthol- induced contraction (10^−3^ M) at 37°C (E_max_ = -8.33 ± 1.22%) was significantly attenuated in the presence of ruthenium red (E_max_ = 2.75 ± 3.60%) and capsaicin (Emax = -1.00 ± 0.62%), but not in the presence of BCTC (Emax = -4, 10 ± 1.01%, Fig 7B).

### Rotundifolone induces an increase in cytosolic Ca^2+^

**Fig 8:**
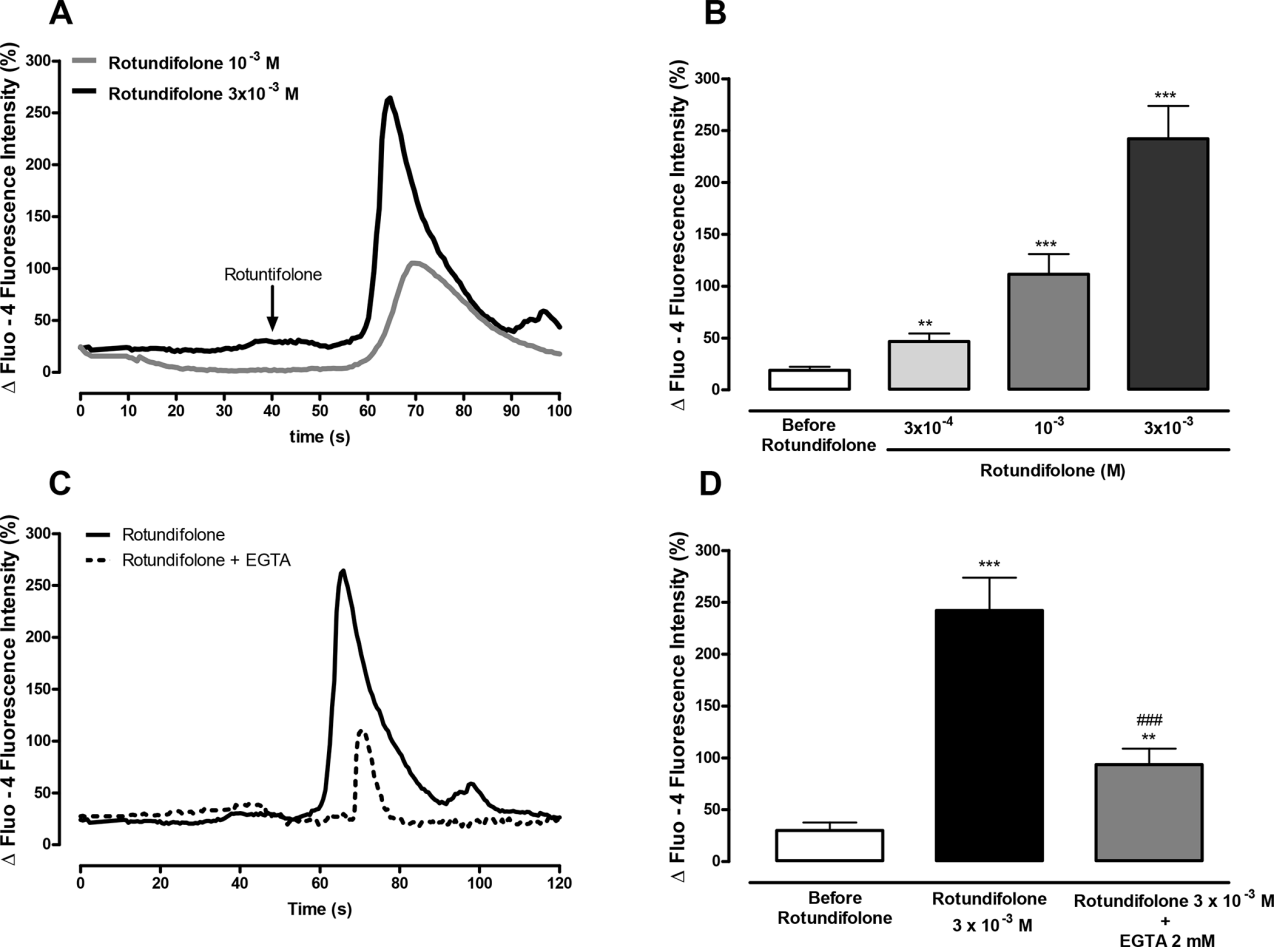
Rotundifolone-induced Ca^2+^ transient in myocytes depend of extracellular Ca^2+^. (A) Representative recording of the Ca^2+^ signals induced by rotundifolone (10^−3^ and 3x10^-3^ M) in freshly dispersed vascular smooth muscle cells from rat superior mesenteric artery. (B) Summary data showing the amplitude of the Ca^2+^ signal induced after perfusion with different concentrations of rotundifolone (3x10^-4^, n = 9; 10^−3^, n = 5; 3x10^-3^ M, n = 11). (C) Representative recording of Ca^2+^ oscillations induced by rotundifolone (3x10^-3^ M) in myocytes placed in Ca^2+^-free medium fortified with 2 mM ethylene glycol tetraacetic acid (EGTA). (D) Summary data demonstrating the decreased amplitude of the Ca^2+^ signal induced by rotundifolone in Ca^2+^-free medium (n = 13). The Ca^2+^ oscillations were measured and represented as increases in fluorescence intensity relative to baseline [DF (%) = (F-F0/F0)*100]. The data were examined using unpaired Student's t tests (rotundifolone with or without EGTA) and one-way ANOVA followed by the Bonferroni post-test. (** p<0.01 and ***p < 0.001 vs control; ###p<0.001 vs Rotundifolone 3x10^-3^ M).

Rotundifolone (3x10^-4^, 10^−3^ and 3x10^-3^ M) significantly increased the fluorescence of Ca^2+^ indicator in freshly dispersed mesenteric artery myocytes, loaded with Fluo-4/AM, in a concentration-dependent manner (46.6 ± 7.8%, n = 9, 111.5 ± 19.3%, n = 5, 242 ± 31.8%, n = 11, respectively) compared to the ​ fluorescence of Ca^2+^ indicator before application of rotundifolone (18.9 ± 3.4%, n = 12). Rotundifolone evoked a rapid and transient increase in intracellular Ca^2+^ levels that was sustained for approximately 25 seconds, as shown in Fig 8A and Fig 2B. This effect was significantly attenuated in myocytes placed in Ca^2+^-free medium fortified with 2 mM EGTA (Fig 8C and 8D).

### Rotundifolone-induced Ca^2+^ signals likely depend on TRPM8 channels

In mesenteric artery myocytes, rotundifolone-induced (3x10^-3^ M) increase in intracellular Ca^2+^ transients was significantly attenuated, but not abolished, following 20 min pretreatment with capsazepine (CPZ 20 μM), a competitive TRPV1 and TRPM8 receptor antagonist [33, 34]. Mean fluorescence of Ca^2+^ indicator in basal conditions, or rotundifolone stimulated, in the presence or absence of CPZ, were: 24.7 ± 5.1% (before rotundifolone administration, n = 17); 564.7 ± 121.0% (p < 0.001 vs control, n = 5), and 121.7 ± 27.5% (p<0.01 vs control and p<0.001 vs Rotundifolone, n = 11), respectively.

## Discussion and Conclusions

The major finding in this study was that the monoterepene, rotundifolone induces vasorelaxation of the superior mesenteric artery through TRPM8 channels. This monoterpene demonstrated a higher selectivity for this channel when compared to menthol. We also demonstrated that TRPM8 channel expression and subsequent activation in isolated rat superior mesenteric artery, signals through vasodilatory pathways.

In previous studies performed by our group, we demonstrated that rotundifolone induced promising relaxation effects in the cardiovascular system. We observed in electrophysiological studies that rotundifolone induces endothelium-independent vasorelaxation in mesenteric artery rings through the activation of BK_Ca_ channels, especially in the lower concentration range (10^−7^ to 10^−4^ M), and through the inhibition of Ca^2+^ entry through L-type Ca^2+^ channels at higher concentrations (10^−4^ to 3x10^-3^ M) [26]. At that point, we had yet to identify the specific target(s) rotundifolone activated nor its mechanism of action. However, it has been reported that a number of monoterpenes act as both agonists and antagonists of different TRP channel family members [35]. Camphor, carvacrol, thymol and menthol have also been shown to activate TRPV3 [36]. Additionally, (-)-Menthol is a well-known agonist for the cold-sensitive channel TRPM8 [37]. Therefore, we hypothesized that rotundifolone targets TRP channels on vascular smooth muscle, altering intracellular Ca^2+^ concentration and consequently activating BK_CA_ channels and thereby leading to inactivation of L-type calcium channel. To confirm this signaling pathway, we performed *in vitro* experiments evaluating the participation of vascular TRPM8 channels in the rotundifolone responses.

The expression of TRPM8 channels in the mesenteric artery has previously been demonstrated. Inoue et al [2] showed a marginal mRNA expression TRPM8 by RT-PCR. Johnson and colleagues [10] have demonstrated TRPM8 protein and mRNA expression in rat mesenteric artery, using RT-PCR and western blotting. In our current study, we investigated the expression of TRPM8 channel in the smooth muscle of isolated mesenteric arteries and observed both TRPM8 channel mRNA as protein expression, corroborating previously published data.

Previous studies have also shown that the TRPM8 channel plays key role in regulating vascular tone and, like other TRP channels, is an important target for monoterpenes [10, 16]. Menthol is widely used as a classical pharmacological activator to study TRPM8 channels [2, 8]. In our study, experiments were performed to compare rotundifolone with menthol, the natural monoterpene origin [16].

Initially, rotundifolone and menthol induced a vasorelaxant effect in isolated superior mesenteric arteries from LN rat, in a concentration dependent manner. Pretreatment with ruthenium red, a non-selective blocker of several subtypes of TRP channels, including TRPV, TRPC3, TRPM6 and TRPA1 [31, 32, 38], but not TRPM8, did not significantly alter rotundifolone-induced relaxation. However, the menthol concentration-response curve was shifted to the right in the presence of ruthenium red. These data suggest that TRP channels blocked by ruthenium red do not participate in the relaxation induced by rotundifolone, however, other TRP channel subtypes, beyond TRPM8, do indeed contribute to cool-mediated/menthol relaxation.

The next experiment, BCTC acting as TRPV1 and TRPM8 channel blockers, were used. We observed that the effect of both rotundifolone and menthol were reduced in the presence of BCTC. The TRPV1 channel blockade, after desensitization with capsaicin, which was applied to rule out the involvement of these channels in the relaxant responses induced by rotundifolone and menthol, demonstrated that capsaicin did not significantly alter the rotundifolone-induced relaxation, but reduced the response induced by menthol at the higher concentrations studied. Together, these data demonstrate that rotundifolone-induced relaxation is more selective for TRPM8 channels than menthol and probably do not involve TRPV1 channels. Additionally, since BCTC not completely block the relaxation induced by rotundifolone and menthol, other relaxing signaling pathways may be involved in the actions of monoterpenes in addition to activation likely to TRPM8 channels. Menthol has previously been reported to have other biological actions independent of TRPM8 activation, including: menthol induced relaxation and inhibited contraction in rat aorta, mesenteric and coronary arteries, primarily through Ca^2+^ influx inhibition, via nifedipine-sensitive Ca^2+^ channels in vascular smooth muscle [39]; menthol induced murine gastric relaxation involving nicotinic receptors, but not TRP and 5-HT3 receptors [40]; menthol induced channel activation and in higher concentrations that lead to a reversible channel block, in whole-cell and single-channel recordings of activity of heterologously expressed TRPA1 channels [41].

Additionally, experiments were performed to investigate the participation of store-operated channels (SOC) in rotundifolone-mediated relaxation. The results indicate that the rotundifolone-induced relaxation was significantly attenuated at 4 different concentrations in the presence of SKF, while having no affect the maximum effect. The relaxation induced by menthol was significantly reduced in all tested concentrations (the only exception being 10^-7^M) suggesting a strong dependence on Ca^2+^ entry through SKF-sensitive channels. TRPM8 have been observed on plasma membrane and sacroplasmic reticulum [42], which may suggest that activation of TRPM8 on sarcoplasmic reticulum membrane, could induce the release of Ca^2+^ and Ca^2+^ store depletion [43–44]. Therefore, SKF 96365-mediated inhibition of menthol and rotundifolone-induced vasorelaxation, may partly be due to the action of monoterpenes on TRPM8 channels in the sarcoplasmic reticulum. However, further studies are needed to evaluate this hypothesis.

TRPM8 channels are classified as thermosensitive TRP channels because they are activated by cold temperatures between the range of 8 to 27°C [45–47]. Additionally, TRPM8 are activated by voltage and cooling compounds, such as menthol. The effects of cold and menthol on TRPM8 have been shown to increase each other [48]. In our studies, vascular tissue was subjected to cold temperatures in order to evaluate the mutual influences of temperature and rotundifolone on TRPM8 channels, as well as evaluate the vascular responses induced by cold temperatures alone in rat mesenteric artery. We observed that decreasing the temperature from 37 to 25° or 18°C induced relaxation of arterial rings. The addition of menthol or rotundifolone in combination with the reduced temperature lead to an increase in vasodilation, which reinforced the participation of TRPM8 channels in vasodilatory pathways in isolated rat mesenteric arteries.

Interestingly, menthol, but not rotundifolone, induced a slight vasoconstriction at 37°C, when added to basal contractile tone, similar to that observed by Johnson and coworkers [10], suggesting that menthol and rotundifolone may not share exact mechanisms of action. Arterial ring pretreatment with ruthenium red or capasaicin did not change the relaxant responses induced by rotundifolone, or menthol, when evaluated at cold temperatures. Moreover, BCTC abolished the relaxant response induced by both menthol and rotundifolone, at both temperatures (25° and 18°C). Taken together, these findings demonstrate various important aspects of rotundifolone pharmacology, as well as TRPM8 channel function in the mesenteric artery. First, our data suggests that the influences of rotundifolone and menthol on TRPM8 channels are related to the BCTC target action site. However, the relaxant response induced solely by cold temperature in mesenteric artery does not seem to be modulated by BCTC (2x10^-6^ M), at least at this concentration tested. Another point worth being emphasized is that the vasoconstriction induced by menthol was blocked after treatment with ruthenium red or capsaicin in rings subjected to 37°C, suggesting that other TRP channels sensitive to these antagonists, are molecular targets for the mechanism of action of menthol, however not to rotundifolone. Furthermore, although BCTC blocks TRPM8 and TRPV1 channels, activation of TRPV1 channels by cold temperatures and consequent relaxation can be ruled out due to the fact that no significant change in the vasorelaxation response of rotundifolone was observed following pretreatment with capsaicin.

TRPM8 channel protein expressed on the plasma membrane has been reported and like most channels TRP, the TRPM8 channel is a cationic channels that allows Ca^2+^ influx across the plasma membrane into the intracellular medium [8, 37]. Given the importance of Ca^2+^ influx that is gated by TRPM8 channels activation, we repeated the experiments with mesenteric rings at cold temperatures in Ca^2+^-free Tyrode´s solution (EGTA 2 mM). Under these conditions, relaxation responses induced only by cold temperature or concurrent treatment with cold temperatures and menthol or rotundifolone were significantly attenuated, suggesting that menthol and rotundifolone are involved in TRPM8 channel activation and Ca^2+^ influx across the plasma membrane.

To directly investigate whether rotundifolone induced Ca^2+^ influx through TRPM8 channels in vascular smooth muscle cells, freshly dispersed myocytes from mesenteric arteries were loaded with Fluo-4/AM to measure the fluorescence of Ca^2+^ indicator. Here, rotundifolone increased cytosolic Ca^2+^ at all concentration tested. Furthermore, EGTA (2 mM) and capsazepin (20 μM), respective TRPV1 and TRPM8 channels antagonist [31], significantly attenuated the changes of cytosolic Ca^2+^ induced by rotundifolone, suggesting that the effects of rotundifolone observed in freshly dispersed mesenteric artery myocytes, indicated the activation of TRPM8 channels, Ca^2+^ influx and functional changes in the myocyte.

In conclusion, based on our previous observations and the data obtained in this study, rotundifolone induces vasorelaxant effects by activating probably TRPM8 channels in rat mesenteric artery, in a manner that was more selectively than menthol. However, additional studies should be conducted to confirm if TRPM8 is the TRP channels subtype involved in vascular relaxation induced by rotundifolone in rat superior mesenteric artery by using knockout animals.

Moreover, consistent with other studies in vascular smooth muscle that demonstrated the importance of TRPV4 channels mediating vasodilation through increases in Ca^2+^ and subsequent activation of BKca channels [49–50], TRPM8 channel activation in rat mesenteric artery appears to signal through a similar pathway. However, more experiments are needed to confirm the functional interaction between TRPM8 channels and BKca channels, in the vascular smooth muscle.

Our study shows that TRPM8 channels are present in rat mesenteric artery and appear to be activated by menthol and rotundifolone, both monoterpenes. Additionally, the TRPM8 channels can contribute to vasomotor tone when activated, which makes them potential targets for pharmacological vasodilators. Moreover, TRPM8 channels may be involved in intracellular Ca2+ control in non-temperature-dependent tissues. Additional studies are needed to assess whether these channels are modified in vascular disorders, such as those seen in hypertension.

## Supporting Information

S1 Supplementary Material Vasorelaxation induced by rotundifolone and Menthol. Relaxants responses induced by rotundifolone (A) and menthol (B) in the presence or absence of functional endothelium. Data are the mean ± SEM (n = 6). The data were examined using unpaired Student's t tests and one-way ANOVA followed by the Bonferroni post-test.
(EPS)
